# Circulating tumor DNA profile and its clinical significance in patients with hormone receptor-positive and HER2-negative mBC

**DOI:** 10.3389/fendo.2022.1075830

**Published:** 2022-11-28

**Authors:** Yu Tang, Jing Li, Binliang Liu, Jialu Ran, Zhe-Yu Hu, Quchang Ouyang

**Affiliations:** ^1^ Department of Breast Cancer Medical Oncology, Hunan Cancer Hospital, Changsha, China; ^2^ The Affiliated Cancer Hospital of Xiangya School of Medicine, Central South University/Hunan Cancer Hospital, Changsha, China; ^3^ Department of Biostatistics and Bioinformatics, Emory University Rollins School of Public Health, Atlanta, GA, United States

**Keywords:** (ctDNA) circulating tumor DNA, metastatic breast cancer, HR-positive and HER2-negative MBC, endocrine therapy, subtype

## Abstract

**Background:**

After early-line (first- and second-line) endocrine therapy, hormone-receptor (HR)-positive and human epidermal growth factor receptor 2 (HER2)-negative metastatic breast cancers (mBCs) become resistant to endocrine therapy. Genetic alterations may underlie resistance to endocrine therapies. This study aims to investigate the circulating tumor DNA (ctDNA) alterations and the clinical implication in hormone-receptor-positive, HER2-negative metastatic breast cancer patients with multiline endocrine therapy failure.

**Methods:**

This registered study (NCT05079074, ClinicalTrials.gov) enrolled 104 patients with hormone-receptor-positive, HER2-negative metastatic breast cancer who progressed after the early-line endocrine therapy. ctDNA alterations were analyzed by next generation sequencing (NGS). ctDNA alterations were ranked and clustered by using R ‘ComplexHeatmap’ and ‘hclust’ function. ctDNA-guided therapy was administrated. Progression-free survival (PFS) was assessed COX regression analysis, and Kaplan-Meier curves were plotted.

**Findings:**

The top ctDNA altered genes were *TP53* (39%), *PIK3CA* (38%), *BRCA1/2* (13%), *ESR1* (12%), *FGFR* (11%), *ERBB2* (11%), and *GATA3* (9%). Among these genes, *TP53*, *PIK3CA* helix domain mutation (*PIK3CA*-HD), *FGFR*, *ESR1* and *GATA3* were related to endocrine therapy resistance. The genetic landscapes changed and tumor mutation burden increased in both *TP53*-altered and *PIK3CA*-altered patients. Both *BRCA1/2* and *ERBB2* alterations correlated with *TP53* alterations (*P*=0.02 and *P*=0.04, respectively). However, while 93% *BRCA1/2* alterations concentrated in *PIK3CA*-wildtype patients, 82% *ERBB2* alterations concentrated in *PIK3CA*-altered patients. Kaplan–Meier curves showed that patients who received druggable ctDNA alteration-guided treatment (DDAT) had significantly longer PFS than those who received physician-chosen therapy, with median PFS of 6.1 months versus 4.6 months (hazard ratio = 0.53, 95% CI: 0.34-0.85, Logrank *P* = 0.006).

**Conclusion:**

Multiple genetic alterations were important reasons for the failure of endocrine therapy for HR-positive and HER2-negative mBC. Targeting these genes might restore the treatment sensitivity and benefit survival.

## Introduction

According to the cancer statistic facts from Surveillance, Epidemiology, and End Results Program (SEER) database, 15% new cancer cases in 2022 are breast cancer cases. The 5-year relative survival for patients with localized, reginal and distant breast cancers were 99.1%, 86.1% and 30.0%, respectively. As a kind of endocrine-related tumor, metastatic breast cancer (mBC) is the leading cause of cancer-related death in women worldwide (1). About 70% of breast cancers are HR-positive and HER2-negative (2). For this group of patients, endocrine therapy (ET) is the most important medical treatment (3). Up to now, the progress in scientific research has prompted us to better understand the pathophysiology of HR-positive and HER2-negative mBCs, so as to develop new drugs to strengthen ET, such as phosphoinositide 3 kinase (PI3K)/mammalian target of rapamycin (mTOR) pathway inhibitors, histone deacetylase (HDAC) inhibitors and cyclin-dependent kinase 4/6 (CDK4/6) inhibitors (4). In clinical practice, the current early-line (the 1^st^ or 2^nd^ line) options include AI/fulvestrant alone or combined with cyclin-dependent kinase 4/6 (CDK4/6) inhibitors. The options for late-line therapy include CDK4/6 inhibitors (for patients who did not receive CDK4/6 in the early-line therapy), phosphoinositide 3 kinase (PI3K)/mammalian target of rapamycin (mTOR) pathway inhibitors, and histone deacetylase (HDAC) inhibitors. The combination of ET and CDK4/6 inhibitors has shown significant survival benefits and is currently considered as the standard first-line regimen (5). With a variety of new therapeutic drugs entering clinical practice, HR-positive and HER2-negative mBC has become a chronic and controllable disease, although it is still incurable (6–12).

However, after a period of treatment, metastatic breast cancer would always develop drug resistance (13). Multiple molecular mechanisms are related to endocrine resistance, including *ESR1* mutation, *FGFR1* amplification, PI3K-AKT or MAPK pathway activation, *etc*. Nuclear protein ER*α*, encoded by *ESR1* gene, is a member of the superfamily of estrogen receptors (ERs) (14). In response to estrogen, ER interacts with the corresponding ER elements (EREs) in nuclear ant subsequently promote cell proliferation. In addition, by cross-talking with membrane tyrosine kinase (TK) receptors, such as HER2 and epidermal growth factor (EGFR), ER plays an important role in the malignant proliferation of breast cancer cells (15). In addition, breast cancer stem cells (BCSCs) attribute to the ET resistance and ER expresses in BCSCs derived from ER-positive BCs (16). ERα36, a variant of ERα36, could rapidly activate MAPKs/ERK pathway and play a pivotal role in anti-estrogen BC resistance (16).

*ERBB2* amplification also reduce the sensitivity of anti-estrogen treatment mainly by activating alternative proliferation signaling pathways, such as PI3K/mTOR pathway and MAPK pathway (17). It has been reported to HER2-activating mutations, which are detected in about 5% of endocrine-resistant mBCs (18), are associated with endogenous and acquired resistance to ET (19). ER-positive breast cancers which have HER2-activating mutants are reported to be resistant to estrogen deprivation and fulvestrant, and also responded poorly to the HER2 tyrosine kinase inhibitor neratinib (19, 20). Therefore, the combined blockade of HER2 and ER have synergistic effects both *in vitro* and *in vivo*. The combination of neratinib with fulvestrant has shown promise in ER-positive mBCs harboring HER2 mutations (21).

In addition, *FGFR1* amplification, which is detected in more than 10% of ER-positive mBCs, is found to be related to ET resistance (22). FGFR1 protein is located on the cell membrane and transduces extracellular signals to promote malignant proliferation. The nuclear FGFR1 can also promote the transcription of ER-targeting genes and cell proliferation; thus, FGFR inhibitor plus fluvestrant show potent anti-tumor effect in ER-positive and FGFR1-amplified cancer cells (22). Besides *FGFR1*, ET-resistance is also found to be related with *FGFR4* amplification and mutations (23). FGFR4 selective inhibitor fisogatinib combined with ET is an attractive strategy (24). In ER-positive mBCs, PI3K pathway components, such as AKT1, PIK3CA, PTEN and mTOR, are frequently aberrant (25). The abnormal activation of PI3K pathway also leads to the acquired resistance to ET (26). Antagonists of the components in PI3K/mTOR pathway are found to improve the treatment outcome of ET, especially for those with tumors harboring activating mutations. For instance, when coupled with fulvestrant, the specific *PIK3CA* inhibitor Alpelisib has shown promising treatment efficiency in recently approved in ER-positive mBCs patients who had received endocrine therapy previously (12).

Our previous has demonstrated that about 60 frequently detected ctDNA alterations can be classified into four major functional subtypes: extracellular function (ECF) subtype, cell proliferation (CP) subtype, nucleus function (NF) subtype and cascade signaling pathway (CSP) subtype (27). In this study, we focused on patients with late-stage HR-positive/HER2-negative metastatic breast cancers who have received the 1^st^ and 2^nd^ line endocrine therapy previously, and used circulating tumor DNA to investigate the ET-resistant biomarkers and the therapeutic targets.

## Methods

### Study design and participants

This study is an observational, multicenter clinical investigation to evaluate the diagnostic and therapeutic values of circulating tumor DNA (cDNA) analysis in patients with metastatic HR-positive/HER2-negative breast cancer. This study enrolled patients (in late-stage mBC) whose metastatic disease progressed after early-line (the first-line and second-line) of ET treatment. Here, late-line therapy defines the third line or even later-line (≥ 3 lines in total) therapy, including ET, chemotherapy, or other targeting therapy. This study was approved by the ethics committee of Hunan Cancer Hospital of Central South University (No. 2017YS031) and registered with ClinicalTrials.gov (NCT05079074). The investigation was carried out in accordance with Chinese laws and regulations and the declaration of Helsinki.

From December 2016 to June 2019, one hundred and four patients with HR-positive/HER2-negative mBC who progressed after at least two lines of ET and are willing to take commercially ctDNA testing from Hunan Cancer Hospital, Changsha fourth hospital and Zhuzhou Hospital were recruited in this study. Eligibility criteria were 1) recent progression of HR-positive/HER2-negative mBC after multiple lines (at least 2 lines) of endocrine therapy; 2) for later-line therapy, there was no available recommendations; 3) score 0 to 2 for Eastern Cooperative Oncology Group (ECOG) performance; 4) the expected survival time is > 3 months; 5) blood routine test, liver and kidney function test meet the following criteria: PLT > 100g/L, Hb > 9g/L, Neutrophil > 2.0g/l; AST and ALT < 2.5 upper limit of normal (ULN); Cr < 1.0ULN; TBIL < 1.5ULN. The exclusion criteria were 1) having multiple primary cancers; 2) unable to obtain blood samples; 3) having a history of organ transplantation or immunodeficiency; 4) serious arrhythmia, abnormal cardiac function or with a history of myocardial infarction. All enrolled patients agreed to participate and signed the consent form.

### Next-generation sequencing and tumor mutation burden analysis

After recent progression of at least 2 lines of endocrine therapy, all recruited patients voluntarily received the ctDNA analyses. According to our previously published method (27, 28), the main procedures of ctDNA testing included blood sample collection, DNA extraction, target capture, next-generation sequencing (NGS), and data analysis. In this study, tumor mutation burden (TMB, number of the somatic mutations per mega-base (Mb)) was calculated from our big ctDNA gene panel (29, 30). TMB analyses interrogated single nucleotide variants (SNVs) and small insertions or deletions (INDELs) with the variant allele frequency (VAF) greater or equal to 3%.

### Heatmap and clustering of ctDNA alterations

In order to rank ctDNA alterations after a recent progression of HR-positive/HER2-negative mBC after multiple lines (at least 2 lines), ‘oncoPrint’ functions from ‘ComplexHeatmap’ package in the R package (version i386 4.1.2, http://www.r-project.org) were applied. Moreover, we used the ‘hclust’ function in ‘ape’ package to perform the hierarchical clustering analysis. ctDNA alterations were clustered by using a set of dissimilarities (31). According to our previous report (27), we used ‘complete’ agglomeration method to cluster, and applied the ‘cutree’ function to prune the clustering results. In the pyramidal clustering tree, the ctDNA alterations on the right subtree had higher clustering scores than those on the left subtree.

### Detailed interventional strategies

After a recent progression of at least 2 lines of endocrine therapy, all recruited patients received ctDNA analyses. Their ctDNA testing reports exhibited that 88/104 (84.6%) patients had ctDNA alterations. Among these ctDNA-positive patients, 62 patients had druggable ctDNA alterations that were clinically relevant. Therefore these 62 patients received druggable ctDNA alteration-guided treatment (DDAT). The remaining 42 patients received physician-chosen late-line therapy. For the patients who received druggable ctDNA alteration-guided treatment (DDAT), patients with ctDNA aberrations in FGFR/VEGFR pathways were treated with anti-FGFR/VEGR inhibitors; patients with ctDNA aberrations in cell cycle pathways were treated with CDK4/6 inhibitor; patients with ctDNA aberrations in homologous recombination repair (HRR) pathway were treated with PARP inhibitor; patients with ctDNA aberrations in PI3K/AKT/mTOR pathway were treated with PI3K/AKT/mTOR pathway inhibitors; patients with ctDNA aberrations in EGFR/HER2 pathway were treated with TKIs or ADCs; patients with ctDNA aberrations in AR or ESR1 were treated with AR antagonists or fulvestrant.

### Treatment response evaluations

Radiology evaluations were performed before the initiation of late-line therapy. Tumor responses were further assessed every two cycles (6 weeks) of late-line therapy and every two treatment cycles (about 6-8 weeks) thereafter by radiology examinations. The treatment response was assessed according to the RECIST 1.1 criterion, as detailed described in our previous reports (27). Progression-free survival (PFS) defined as the survival time between the beginning of late-lint therapy to death or the progression. The patients’ death information was inaccessible due to the security reasons. For PFS analysis, the date of last visit for non-progressive patients was censored.

### Statistical analysis

In this study, Kaplan–Meier curves coupled with logrank test was used to assess the effect of druggable ctDNA alteration-guided treatment (DDAT) on the PFS. The Cox proportional hazards model was used to estimate the treatment effect which was presented as the hazard ratio and 95% confidence interval (CI). All statistical analyses were conducted by using SAS 9.4 software (SAS Institute Inc., NC, USA) and R 4.1.2 (https://www.r-project.org/). All hypothesis tests were two sided and conducted at a significance level of 0.05.

## Results

### Patient characteristics

This study recruited 104 eligible late-stage HR-positive/HER2-negative mBC patients. All these patients progressed after early-line (at least two lines) ET therapy and then received ctDNA testing. The patient characteristics were listed in **Table 1**. The age of study population ranged from 28 to 70 years, with the median age of 48 years. More than 95% patients were younger than 65 years. The ECOG performance was 0 in 51 (49%) patients and 1 in 53 (51%) patients. 29 (27%) patients had bone-only metastases. 12 (11.5%) patients had distant lymph node metastases or soft tissue metastases. 63 (60.6%) patients had visceral metastases. 78 (75%) patients had ER-positive and PR-positive disease, 18 (17.3%) patients had ER-positive and PR-negative disease and 8 (7.7%) patients had ER-negative and PR-positive disease. A total of 58 (55.8%) patients had HER2(0) disease and 46 (44.2%) patients were HER2-low (1+ or 2+) disease. 87 (83.7%) had invasive carcinoma (ductal or lobular).

**Table 1:** Patient characteristics of late-stage HR-positive, HER2-negative metastatic breast cancers.

Characteristics	Total (n=104)	
Age (years)		
Median (IQR)	48	(41-56)
Range		(28-70)
< 65	99	(95.2)
≥ 65	5	(4.8)
Eastern Cooperative Oncology Group performance status, n (%)		
0	51	(49.0)
1	53	(51.0)
Metastasis sites^#^, n (%)		
Visceral (with/without bone)	63	(60.6)
Soft tissue (without visceral mets)	12	(11.5)
Bone only	29	(27.9)
ER/PR, n (%)		
ER positive and PR positive	78	(75.0)
ER positive and PR negative	18	(17.3)
ER negative and PR positive	8	(7.7)
HER2, n (%)		
0	58	(55.8)
1+	30	(28.8)
2+ (FISH-negative)	16	(15.4)
Pathological type, n (%)		
Invasive ductal carcinoma	66	(63.5)
Invasive lobular carcinoma	7	(6.7)
Invasive carcinoma	14	(13.5)
Other	17	(16.3)
First-line endocrine therapy		
AI	21	(20.2)
AI + CDK4/6 inhibitor	30	(28.8)
Fulvestrant	24	(23.1)
Fulvestrant + CDK4/6 inhibitor	18	(17.3)
Tamoxifen/Toremifen	4	(3.8)
Other*	7	(6.7)
Second-line endocrine therapy		
AI	23	(22.1)
AI + CDK4/6 inhibitor	11	(10.6)
Fulvestrant	21	(20.2)
Fulvestrant + CDK4/6 inhibitor	28	(26.9)
Tamoxifen/Toremifen	10	(9.6)
Other	11	(10.6)

For metastasis sites^#^, patients were categorized to visceral metastases (with/without soft tissue mets or bone mets), soft tissue metastases (with/without bone mets and without visceral mets) and bone-only metastases.

Other* represents the first- or second-line use of endocrine therapy, including AI/Fulvestrant/Tamoxifen/Toremifen combined with Evelimus or Chidamide.

ER (Estrogen Receptor), PR (Progesterone Receptor), HER2 (Human Epidermal Growth Factor Receptor-2).

### Circulating tumor DNA alterations profiles

**Figure 1:**
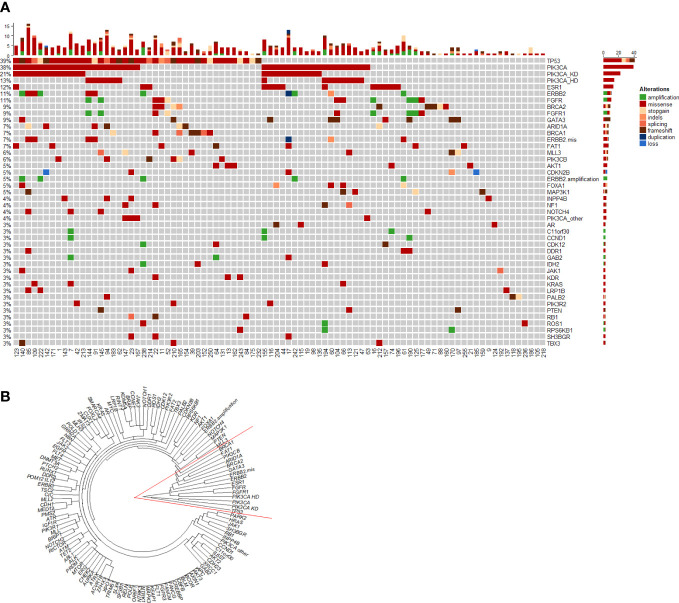
Heatmap and cluster of ctDNA alterations. **(A)** Heatmaps of ctDNA alteration profiles for patients with metastatic ER-positive and HER2-negative breast cancer. **(B)** Circle plot of ctDNA alterations among 104 patients. Genes were clustered by the ‘complete’ method of the hclust function in R.

**Figure 2:**
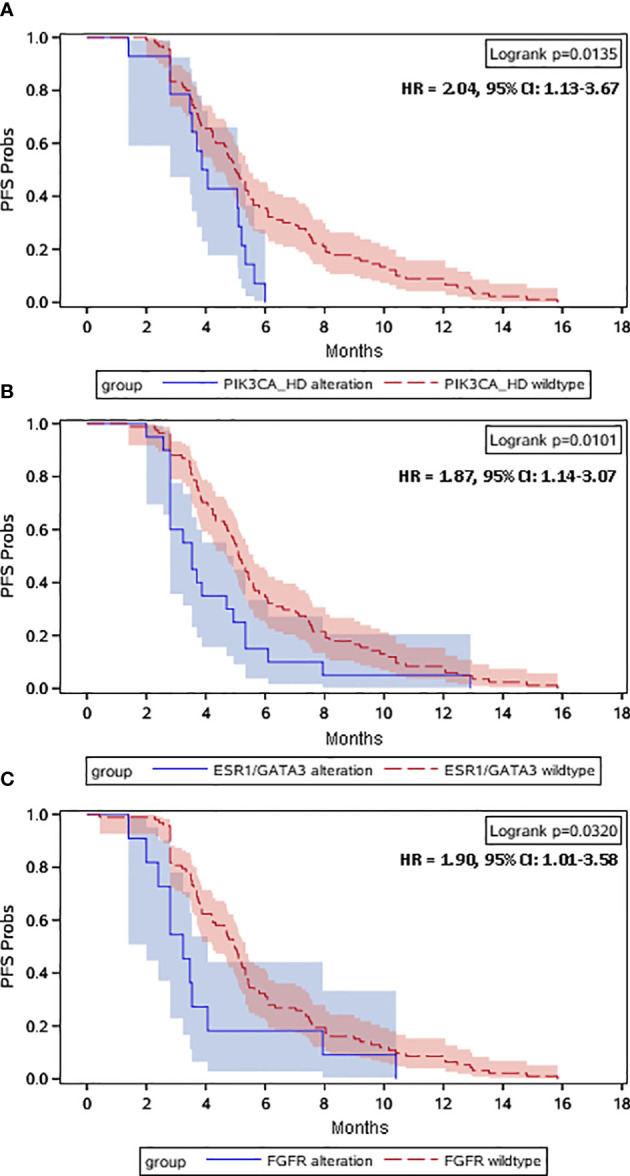
Kaplan–Meier plots of PFS in prior-line treatment. Dashes represent censored patients. HR, hazard ratio. Univariate Cox regression analysis was performed to calculate the hazard ratio (HR) with 95% confidence interval (CI) of progression in the prior-line treatment for metastatic HR-positive/HER2-negative breast cancer patients with *PIK3CA*-HD alterations versus with *PIK3CA* wild-type **(A)**, *ESR1*/*GATA3* alterations versus *ESR1*/*GATA3* wild-type **(B)** and *FGFR* alterations versus *FGFR* wild-type **(C)**.

When the disease progressed after the early line (≤2 lines) endocrine therapy, ctDNA alterations were evaluated for all the recruited HR-positive/HER2-negative mBC patients. **Figure 1A** heatmap exhibited that a total of 88 (85%) patients had ctDNA alterations. The top ctDNA altered genes were *TP53* (39%), *PIK3CA* (38%), *BRCA1/2* (13%), *ESR1* (12%), *FGFR* (11%), *ERBB2* (11%), and *GATA3* (9%). All these altered genes were clustered in the right red angle of the cluster tree (**Figure 1B**). Among these genes, *PIK3CA-HD*, *ESR1/GATA3* and *FGFR* were important endocrine therapy resistant genes. Kaplan-Meier curves were plotted to compare the progression-free survival (PFS, **Figure 2**). *TP53* alteration was not a risk factor for endocrine therapy resistance. The risk for progression among patients with *PIK3CA-HD* was significantly higher than that among *PIK3CA-WT* patients (hazard ratio: 2.04, 95% CI: 1.13-3.67, Logrank *P*=0.0135, **Figure 2A**). The risk for progression among patients with *ESR1/GATA3* alteration or *FGFR* alteration was marginally higher than that among patients with *ESR1/GATA3-WT* (hazard ratio: 1.87, 95% CI: 1.14 -3.07, Logrank *P*=0.01, **Figure 2B**) or patients with *FGFR-WT* patients (hazard ratio: 1.90, 95% CI: 1.01-3.58, Logrank *P*=0.032, **Figure 2C**), respectively.

### Circulating tumor DNA alterations with distinct genetic landscapes

**Figure 3:**
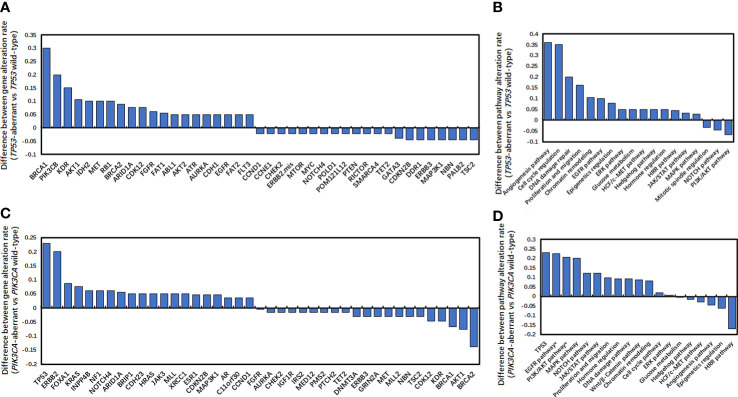
Difference of gene alteration rates and pathway alteration rates. **(A, B)** Difference of gene alteration rates **(A)** and pathway alteration rates **(B)** between *TP53*-aberrant and *TP53*wild-type metastatic HR-positive and HER2-negative breast cancers. **(C, D)** Difference of gene alteration rates **(C)** and pathway alteration rates **(D)** between *PIK3CA* -aberrant and *PIK3CA* wild-type metastatic HR-positive and HER2-negative breast cancers.

It has been demonstrated that compared with *TP53* wild-type metastatic triple-negative breast cancers (mTNBCs), *TP53*-aberrant mTNBCs had significantly higher TMB (32). **Figure S1A** also showed that compared to *TP53* wild-type HR-positive/HER2-negative mBCs, *TP53*-aberrant HR-positive/HER2-negative mBCs also had significantly higher TMB (6.08 muts/Mb *vs* 4.93 muts/Mb; *P <*0.01). In addition, *TP53*-aberrant and *TP53* wild-type HR-positive/HER2-negative mBCs had distinct genetic landscapes. Gene aberrations in angiogenesis, cell cycle regulation, DNA damage repair, proliferation and migration, chromatin remodeling, EGFR pathway were strongly associated with *TP53* aberrations (**Figures 3A, B**).

Moreover, *PIK3CA*-aberrant mBCs also had significantly higher TMB than *PIK3CA* wild-type mBCs (8.77 muts/Mb *vs* 3.14 muts/Mb; *P* < 0.001) (**Figure S1B**). In addition, *PIK3CA*-aberrant and *PIK3CA* wild-type HR-positive/HER2-negative mBCs also showed had different gene aberrations and pathway enrichment. Gene aberrations in EGFR, PI3K/AKT, MAPK, NOTCH and JAK/STAT pathways were strongly associated with *PIK3CA* aberrations (**Figures 3C, D**). These data suggested that both *TP53* and *PIK3CA* alterations could lead to genomic instability and significantly increased tumor mutation loads in HR-positive/HER2-negative mBCs.

*BRCA1/2* and *ERBB2* alterations were also commonly detected in HR-positive/HER2-negative mBCs. **Table S1** showed that, compared to *BRCA1/2*-wildtype patients, more *BRCA1/2*-altered patients were *PIK3CA* wildtype (57.78% *vs* 92.86%, *P*=0.02) and had *TP53* alteration (64.29% *vs* 35.56%, *P*=0.04). *ERBB2* is another important altered gene in late-stage HR-positive/HER2-negative mBCs. Compared to *ERBB2*-WT patients, patients with *ERBB2* alterations had more *PIK3CA* alterations (81.82% *vs* 32.26%, P=0.002, **Table S1**). These findings suggested that *BRCA1/2* alteration were correlated with *TP53* alteration in late-stage HR-positive/HER2-negative mBCs. Unlike *BRCA1/2* alterations which were correlated with *PIK3CA* wildtype, *ERBB2* alterations were correlated with *PIK3CA* alterations.

### Circulating tumor DNA subtypes and late-line therapy

**Figure 4:**
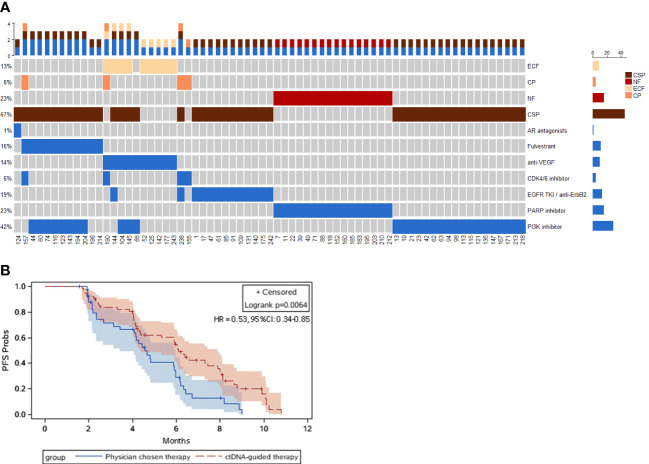
ctDNA subtype and ctDNA-guided lateline therapy. **(A)** Heatmaps of ctDNA subtypes and the corresponding treatment strategies for patients with metastatic HR-positive and HER2-negative breast cancer. **(B)** Kaplan–Meier plots of PFS in late-line treatment. Dashes represent censored patients. HR, hazard ratio. Univariate Cox regression analysis was performed to calculate the hazard ratio (HR) with 95% confidence interval (CI) of progression in the ctDNA-guided therapy group versus the physician chosen therapy group.

Our previous study has demonstrated four ctDNA alteration-based subtypes, extracellular function (ECF), cell proliferation (CP), nuclear function (NF), and cascade signaling pathway (CSP), according to clustering scores and functions (27). Here, 69 (66.35%) patients received druggable ctDNA alteration-guided treatment (DDAT). Heatmap. **Figure 4A** exhibited that, in late-stage HR-positive/HER2-negative mBCs, most cases (67%) concentrated in CSP subtype and received EGFR TKI/anti-ERBB2 treatment, PI3K inhibitor, fulvestrant, or AR antagonists. 23% patients were NF subtype, 13% patients were ECF subtype, and 6% patients were CP subtype. Some patients had ctDNA alterations in two subtypes and received combined targeting treatment. Druggable ctDNA alteration-guided treatment (DDAT) included EGFR TKI/anti-ERBB2 treatment, PI3K inhibitor, fulvestrant, AR antagonists, CDK4/6 inhibitor, PARP inhibitor and anti-VEGF therapy (**Figure 4A**).

OlympiAD study had showed that HER2-negative mBC patients with *BRCA1/2* mutations would be benefited from PARP inhibitor (PARPi) 33). In this study, 14/104 (13%) late-stage HR-positive/HER2-negative mBC patients had *BRCA1/2* alterations and received PARPi treatment. The median PFS of patients who received PARPi was 7.5 months (95% CI: 6.3-9.7); whereas the median PFS of patients without PARPi was 4.8 months (95% CI: 4.2-6.0). The risk for progression of patients with PARPi was significantly lower than the patients without PARPi (hazard ratio: 0.44, 95% CI=0.23~0.86, Logrank *P* =0.01, **Figure S2**). In addition, Kaplan–Meier curves were generated to compare the PFS of patients who received druggable ctDNA alteration-guided treatment (DDAT) with that of those who received physician-chosen therapy. The median PFS of patients with physician-chosen therapy was 4.6 months (95% CI: 3.4-5.0); whereas the median PFS of patients who received druggable ctDNA alteration-guided therapy was 6.1 months (95% CI: 4.4-7.9), with a hazard ratio of 0.53 (95% CI: 0.34-0.85, Logrank *P* = 0.006, **Figure 4B**).

## Discussion

For HR-positive/HER2-negative mBCs after multiple line treatment failure, there is no evidence-based guidelines for late-line therapy. ctDNA testing might provide the genetic alteration information for targeting therapy. Also, the genetic alterations may underlie resistance to endocrine therapy, and strategies may be developed to address such treatment resistance. ctDNA subtypes are more representative than HR/HER2 subtype for late-line therapy. 33). Our previous study has demonstrated four ctDNA-alteration-based subtypes, extracellular function (ECF), cell proliferation (CP), nuclear function (NF), and cascade signaling pathway (CSP). The corresponding druggable ctDNA alteration-guided late-line therapies included anti-VEGF/FGFR therapy, CDK4/6 inhibitor, PARP inhibitor, fulvestrant, AR antagonists, EGFR-TKI/anti-ERBB2 treatment, and PI3K/mTOR inhibitors.

OlympiAD has demonstrated the benefit of Olaparib in HER2-negative mBC patients who had previously received at least one-line prior endocrine therapy (33). However, in HR-positive/HER2-negative mBC subgroup analysis, Olaparib treatment reached an ORR of 65.4%, but without a PFS improvement (hazard ratio 0.82, 95% CI 0.55–1.26) (34). In EMBRACA study, Talazoparib monotherapy also significantly improved the PFS, compared to physician-chosen chemotherapy (35). In this study, 91% of HR-positive/HER2-negative mBC patients had been previously received endocrine therapy. Notably, the self-reported-outcome highlighted significant improvements of health status in patients with PARPi (36). An observational prospective cohort LUCY reported that the median PFS in HR-positive/HER2-negative mBC patients was 8.3 months (95% CI 7.60–9.80) in PARPi treatment group. In addition, BROCADE3 trial assessed the effects of Veliparib and showed that Veliparib prolonged the absolute PFS of 1.9 months (hazard ratio 0.71, 95% CI 0.57–0.88, median PFS 14.5 versus 12.6 months) (37). In HR-positive/HER2-negative mBC patients, the risk of progression was decreased by 31% (hazard ratio 0.69, 95% CI 0.52–0.92). All these results clarified the efficiency of PARPi in BRCA1/2 mutant and HR-positive/HER2-negative mBC patients.

In patients with early-stage ER-positive breast cancers, *PIK3CA* mutations are reported to be associated with a favor prognosis (38, 39). Nonetheless, in HR-positive mBC patients, particularly for whose tumors carrying activating *PIK3CA* mutations, PI3K antagonists could improve the treatment outcome. For instance, capivasertib (AKT inhibitor) combined with fulvestrant, has shown preliminary efficacy in endocrine-resistant ER-positive breast cancers, especially in tumors with *AKT1* mutations (40). When combined with fulvestrant, the specific inhibitor of PI3Kα (encoded by *PIK3CA*), alpelisib, has been approved to improve the treatment outcome (12). In addition, regardless of *PIK3CA* mutational status, everolimus (mTORC1 inhibitor) coupled with aromatase inhibitors (Ais) has been approved for mBC patients who have progressed after ET (6, 41).

Besides PI3K/AKT pathway, FGFR emerged as a new therapeutic target. The amplification of *FGFR* gene was associated with the resistance to endocrine therapy and to CDK4/6 inhibitors (42). Phase I trial (NCT03238196) demonstrated that the triplet combination of erdafitinib (FGFR inhibitor), palbociclib and fulvestrant in HR-positive/HER2-negative, *FGFR*-amplified metastatic breast cancer patients who had one line of therapy in the metastatic setting including prior palbociclib use.

The current study focused on late-line HR-postive/HER2-negative mBCs. Patients with *PIK3CA* helix domain mutation (*PIK3CA*-HD), *FGFR*, *ESR1* and *GATA3* had poor treatment outcome in early-line endocrine therapy. *PIK3CA*-HD, *FGFR*, *ESR1* and *GATA3* were important reasons for the failure of endocrine therapy for HR-positive/HER2-negative mBCs. The genetic landscapes changed and tumor mutation burden increased in both *TP53*-altered and *PIK3CA*-altered patients. By targeting these genes, druggable ctDNA alteration-guided treatment (DDAT) could restore the treatment sensitivity and benefit late-stage patients.

To address a soon to come critical question in patients who progressed after the 1^st^ and 2^nd^ line endocrine therapy, this study tried to evaluate whether ctDNA guided late line therapy might be more beneficial than conventional physician chosen therapy. This study detailed ctDNA alteration profile and ctDNA guided therapy. Overall, this is a very important study highlighting the utility for ctDNA to guide patient therapy in HR-positive and HER2-negative mBCs.

## Data availability statement

The datasets presented in this study can be found in online repositories. The names of the repository/repositories and accession number(s) can be found below: We used NGS sequencing data from the private Geneplus database and from hospitals. Geneplus sequencing data will be made available upon reasonable request.

## Ethics statement

The study was approved by the Ethics Committee at the Hunan Cancer Hospital/The Affiliated Cancer Hospital of Xiangya School of Medicine, Central South University. The patients/participants provided their written informed consent to participate in this study.

## Author contributions

QO, Z-YH and YT were responsible for the verification of the underlying data. QO, Z-YH, BL and JR were responsible for conception, study design, data analysis, interpretation and manuscript writing. YT, JL, BL and QO were responsible for the provision of study materials or patients. YT, JL and BL were responsible for the collection and assembly of data. All authors were responsible for the final approval of the manuscript. All authors contributed to the article and approved the submitted version.

## Funding

This work was supported by Pfizer GMG-2021-ONC-L_China Medical Education Project “A multicenter cross-sectional real-world study of HR-positive and HER2-negative advanced breast cancer patients in Hunan Province”, Hunan Cancer Hospital Climb Plan, Key Grants of Research and Development in Hunan Province (2018SK2124, 2018SK2120 and 2019SK20322), Hunan Provincial Health Commission Project (B2019089, C2019070). The fundings support the whole project, including the study design, data acquisition, data analysis and manuscript preparation. QO had the full access to dataset and the decision to submit for publication.

## Acknowledgments

We acknowledged all the patients from Hunan Cancer Hospital, Changsha fourth hospital and Zhuzhou Hospital Affiliated to Xiangya Medical College of Central South University who agreed to participate in this study. We acknowledge Hunan Medical Association for the management of the Pfizer GMG-2021-ONC-L_China Medical Education Project “A multicenter cross-sectional real-world study of HR-positive and HER2-negative advanced breast cancer patients in Hunan Province”.

## Conflict of interest

The authors declare that this study received funding from Pfizer (GMG-2021-ONCL_ China Medical Education Project “A multicenter cross-sectional real-world study of HR-positive and HER2-negative advanced breast cancer patients in Hunan Province”). The funder had the following involvement with the study: whole project support, study design, data acquisition, data analysis and manuscript preparation.

## Publisher’s note

All claims expressed in this article are solely those of the authors and do not necessarily represent those of their affiliated organizations, or those of the publisher, the editors and the reviewers. Any product that may be evaluated in this article, or claim that may be made by its manufacturer, is not guaranteed or endorsed by the publisher.

## Supplementary material

Supplementary Figure 1 Tumor mutation burden (TMB) in late-stage HR-positive/HER2-negative metastatic breast cancers. **(A)** Difference of TMB between *PIK3CA*-aberrant and *PIK3CA* wild-type metastatic HR-positive and HER2-negative breast cancer **(B)** Difference of TMB between *TP53*-aberrant and *TP53* wild-type metastatic HR-positive and HER2-negative breast cancer.

Supplementary Figure 2 Kaplan–Meier plots of PFS in late-line treatment. Dashes represent censored patients. HR=hazard ratio. Univariate Cox regression analysis was performed to calculate the hazard ratio (HR) with 95% confidence interval (CI) of progression in the PARPi group versus the non-PARPi group.
