# Supplementary figures and images for: Circulating tumor DNA profile and its clinical significance in patients with hormone receptor-positive and HER2-negative mBC
Source: Front Endocrinol (Lausanne). 2022 Nov 28;13:1075830. doi: 10.3389/fendo.2022.1075830 (PMC9742482; doi:10.3389/fendo.2022.1075830)

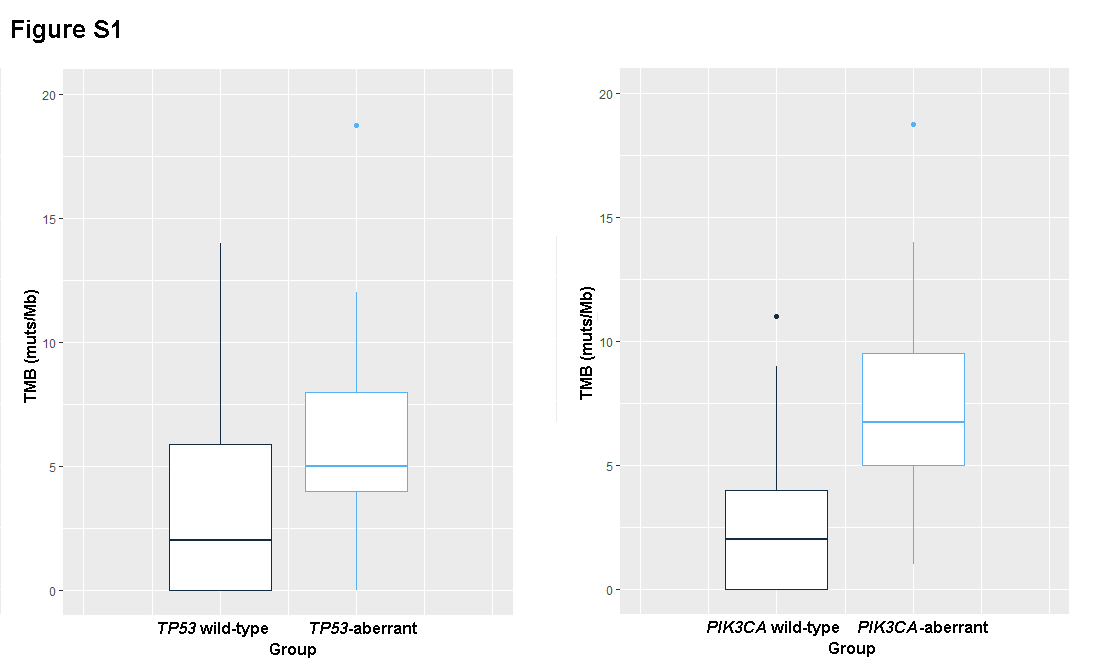

Supplement: Supplementary Figure 1 — Tumor mutation burden (TMB) in late-stage HR-positive/HER2-negative metastatic breast cancers. (A) Difference of TMB between PIK3CA-aberrant and PIK3CA wild-type metastatic HR-positive and HER2-negative breast cancer (B) Difference of TMB between TP53-aberrant and TP53 wild-type metastatic HR-positive and HER2-negative breast cancer. [file Image_1.tif]

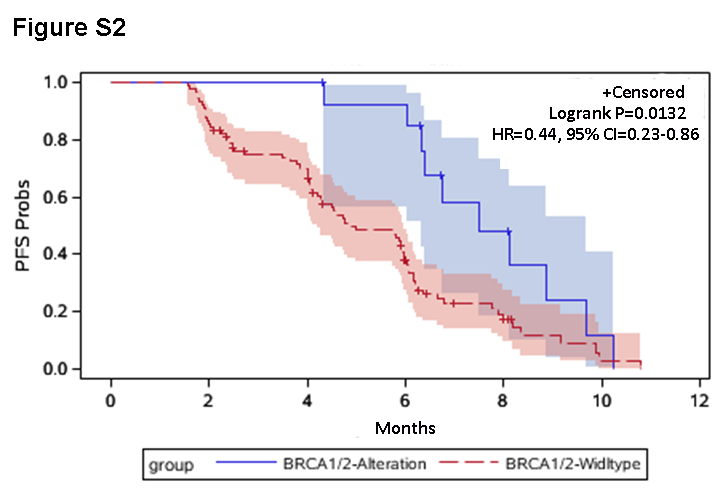

Supplement: Supplementary Figure 2 — Kaplan–Meier plots of PFS in late-line treatment. Dashes represent censored patients. HR=hazard ratio. Univariate Cox regression analysis was performed to calculate the hazard ratio (HR) with 95% confidence interval (CI) of progression in the PARPi group versus the non-PARPi group. [file Image_2.tif]
