# Supplementary material for: Circulating tumor DNA profile and its clinical significance in patients with hormone receptor-positive and HER2-negative mBC
Source: Front Endocrinol (Lausanne). 2022 Nov 28;13:1075830. doi: 10.3389/fendo.2022.1075830 (PMC9742482; doi:10.3389/fendo.2022.1075830)
Supplement: Supplementary file 3 [file Table_1.docx]

**Table S1. ctDNA alterations in late-stage HR-positive, HER2-negative metastatic breast cancers.**

|  | ***PIK3CA*** | | |  | **TP53** | | |
| --- | --- | --- | --- | --- | --- | --- | --- |
|  | **Wildtype (n=65)** | **Alteration (n=39)** | **p-value*** |  | **Wildtype (n=63)** | **Alteration (n=41)** | **p-value*** |
| ***BRCA1/2*** |  |  | 0.02 |  |  |  | 0.04 |
| **wildtype** | 52 (57.78%) | 38 (42.22%) |  |  | 58 (64.44%) | 32 (35.56%) |  |
| **alteration** | 13 (92.86%) | 1 (7.14%) |  |  | 5 (35.71%) | 9 (64.29%) |  |
| ***ESR1/GATA3*** |  |  | 0.44 |  |  |  | 0.14 |
| **wildtype** | 54 (64.29%) | 30 (35.71%) |  |  | 48 (57.14%) | 36 (42.86%) |  |
| **alteration** | 11 (55.00%) | 9 (45.00%) |  |  | 15 (75.00%) | 5 (25.00%) |  |
| ***FGFR*** |  |  | 1.00 |  |  |  | 0.67 |
| **wildtype** | 58 (62.37%) | 35 (37.63%) |  |  | 57 (61.29%) | 36 (38.71%) |  |
| **alteration** | 7 (63.64%) | 4 (36.36%) |  |  | 6 (54.55%) | 5 (45.45%) |  |
| ***ERBB2*** |  |  | 0.002 |  |  |  | 0.11 |
| **wildtype** | 63 (67.74%) | 30 (32.26%) |  |  | 59 (63.44%) | 34 (36.56%) |  |
| **alteration** | 2 (18.18%) | 9 (81.82%) |  |  | 4 (36.36%) | 7 (63.64%) |  |

Note: * p-value was calculated by Chi-square test, or Fisher’s exact test (when sample size ≤ 5) for ctDNA alteration categorical variables.
